# Oral rinses in growth inhibition and treatment of *Helicobacter pylori* infection

**DOI:** 10.1186/s12866-020-01728-4

**Published:** 2020-03-04

**Authors:** Dharmendra Kashyap, Budhadev Baral, Tarun Prakash Verma, Charu Sonkar, Debi Chatterji, Ajay Kumar Jain, Hem C. Jha

**Affiliations:** 1 Discipline of Biosciences and Biomedical Engineering, Indian Institute of Technology Indore, 453552 Indore, Madhya Pradesh India; 2 Choithram Hospital and Research Centre, 452014 Indore, Madhya Pradesh India

**Keywords:** Gastric cancer, Oral rinses, CagA, Apoptotic pathway, Growth curve, *H. pylori*, Gastritis

## Abstract

**Background:**

*Helicobacter pylori* (*H. pylori*) is well-known for its role in chronic gastritis and gastric cancer. Eradication of these carcinogenic bacteria from the gut is one of the challenges for clinicians. The complexity of treatment mainly owes to antibiotic resistance and relapse due to an additional reservoir in the oral cavity. Our study emphases the isolation of *H. pylori* from distinct habitats of the gut microenvironment (gastric biopsy and gastric juice) and its subsequent characterization. We have also evaluated the effect of various oral rinses on isolated *H. pylori* from different anatomical locations of included subjects.

**Results:**

The possible strains isolated from two different habitats of the same subject shows a striking difference in their growth pattern. Promisingly, some of the included oral rinses are efficient in growth inhibition as per recommended 30 s treatment. The subsequent evaluation shows that oral rinse B (among A-E) is most effective and down-regulates the expression of one of the potent *H. pylori* gene, CagA, in the infected gastric adenocarcinoma (AGS) cells.

**Conclusion:**

Our study, for the first time, revealed that *H. pylori,* isolated from the different habitat of the same subject, show a different growth pattern. The expression of *H. pylori* pathogenic gene (CagA) was down-regulated by the use of oral rinses. Hence, oral rinses will reduce the *H. pylori* in the oral cavity and help to control its migration from oral to the gastric compartment and may be used as an adjuvant treatment option for its re-infection.

## Background

*Helicobacter pylori* play a vital role in the development of various gastro-duodenal diseases [1]. Usually, healthy microflora produces a bacteriocin-like inhibitory protein that inhibits *H. pylori* growth [2]. The number of *H. pylori* may increase due to loss in healthy microflora. Subsequently, it leads to the production of gastric acid, followed by ulceration [3]. Some strains of *H. pylori* are virulent, and host factors may also be responsible for disease progression [4]. Additionally, other bacteria that are acid-tolerant might also reside at the infection site within ulcers and thus enhance the problem caused by *H. pylori* [5].

Worldwide, *H. pylori* have been classified according to population genetics tool (STRUCTURE) developed by Pritchard et al. [6, 7]. Broadly, they represent geographical areas and named as*: hpEurope, hpSahul, hpEastAsia, hpAsia2, hpNEAfrica, hpAfrica1* and *hpAfrica2* [8]. Also, there is a large variability in the occurrence of gastric cancer (GC) worldwide [9]. Asian countries such as South Korea, China, and Japan have a high incidence of GC [10]. India is a low-risk country for GC; however, it may be attributed to underreporting [11]. There lies a lacuna in the epidemiological studies and reporting from small towns and villages, which represents a large part of the Indian population [12].

*H. pylori* seroprevalence in the adult population of developing countries varies from 55 to 92%. In contrary to this, the seroprevalence of *H. pylori* in Chinese and Japanese adults is 44 and 55%, respectively [13]. The primary manifestation of *H. pylori* in India is the duodenal ulcer, which is a major concern [14]. A study suggested that 56% of *H. pylori* infection contributes to the leading cause of GC [15]. Therefore, a comprehensive study of *H. pylori* strains and its pathogenic properties is crucial in the Indian scenario.

*H. pylori* may be transmitted through an oral-oral or oro-fecal route, and thus oral cavity may act as its possible reservoir [16]. Its presence in the oral cavity is seldom eliminated by *H. pylori* eradication therapy [17]. Moreover, the oral site may act as a source for re-infection, which is found to be as high as 60% in Indian subjects [18, 19]. Hence, its eradication from oral microenvironment is essential [20, 21]. Several antimicrobial (e.g., bisbiguanides, metal ions, phenols and quaternary ammonium compounds) and antiplaque agents (e.g., surfactants and essential oils) in the form of toothpaste and mouth rinses have been formulated [22]. Antiplaque agents destroy bacterial biofilm, which prevents adherence and growth of bacteria, while antimicrobial agents inhibit the growth or kill the target bacteria [23].

Importantly, many virulent strains of *H. pylori* harbor numerous adhesins (BabA/B, SabA, AlpA/B, OipA and HopZ) and the *cag* (cytotoxin-associated genes) pathogenicity island encoding a type IV secretion system (T4SS) [24]. A tight bacterial contact with the host cell may get established by the adhesins [25]. Moreover, bacterial effector proteins like CagA is delivered into host cells through this secretion system [26]. A study also mentioned that *H. pylori* colonization might also depend on the alteration of mucosal epithelial apoptosis by chronic inflammation [27]. Surprisingly, the relation between various *H. pylori* serotypes, their growth alone, or with gastric epithelial microenvironment in order to the possible occurrence in GC has not been evaluated to date. Investigation of *H. pylori* strains for their growth, and subsequent host cell transformation ability may open better understanding in this domain. Hence, the present study was designed to evaluate the growth pattern of various clinical isolates of *H. pylori* and their response to treatment with commercially available oral rinses/solution. The study also includes the evaluation of tumor suppressor and proto-oncogene status in gastric epithelial cells as a response to treated and untreated isolates of *H. pylori*.

## Results

### Gastric biopsy and juice collection from gastritis patients for isolation of *H. pylori*

**Fig. 1:**
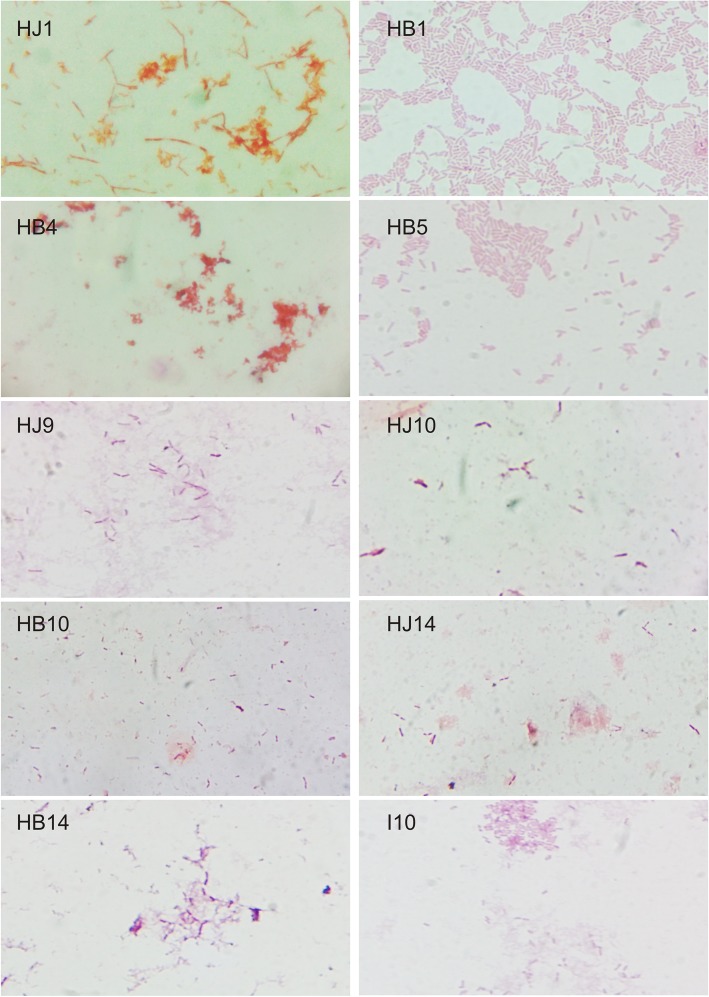
Identification of bacteria through Gram staining. Gram staining of different clinical isolates of *H. pylori,* namely, I10, HJ9, HB10, HJ10, HB14, HJ14, HB1, HJ1, HB4, and HB5 were showing typical gram-negative bacteria

To date, there are no *H. pylori* isolates reported from central India to the best of our knowledge. Moreover, isolates from northern and southern India have been listed in previous reports [28]. *H. pylori* were successfully isolated from five out of 14 biopsy samples and four out of 11 juice samples (Table 1). After observation in antibiotic selective media, Gram staining was performed on all isolates (Fig. 1). Further, three clinical isolates from biopsies and one from juice were subjected for the amplification of *H. pylori* (16 s rRNA) through qRT-PCR (data not shown). Additionally, validation of the clinical isolates was confirmed through nucleotide sequencing (data not shown). Furthermore, the growth of bacteria may be attributed to its pathogenic ability; hence we have studied the growth pattern of the isolated *H. pylori* and compared it with the reference strain (I10).

**Table 1:** Gastric biopsy and juice collection from gastritis patients

Sr. No.	Sample		Sex	RUT status	Grown sample	
	HB	HJ				
1	✓	✓	F	+	HB1	HJ1
2	✓	✓	M	+	–	–
3	✓	✓	M	+	–	–
4	✓	✘	M	+	HB4	–
5	✓	✘	M	+	HB5	–
6	✓	✘	M	+	–	–
7	✓	✓	M	+	–	–
8	✓	✓	F	+	–	–
9	✓	✓	F	+	–	HJ9
10	✓	✓	M	+	HB10	HJ10
11	✓	✓	M	+	–	–
12	✓	✓	M	+	–	–
13	✓	✓	F	+	–	–
14	✓	✓	F	+	HB14	HJ14

*RUT* Rapid Urease Test, *HB* Gastric Biopsy, *HJ* Gastric Juice, *M* Male, *F* Female

### The growth pattern of different clinical isolates of *H. pylori*

**Fig. 2:**
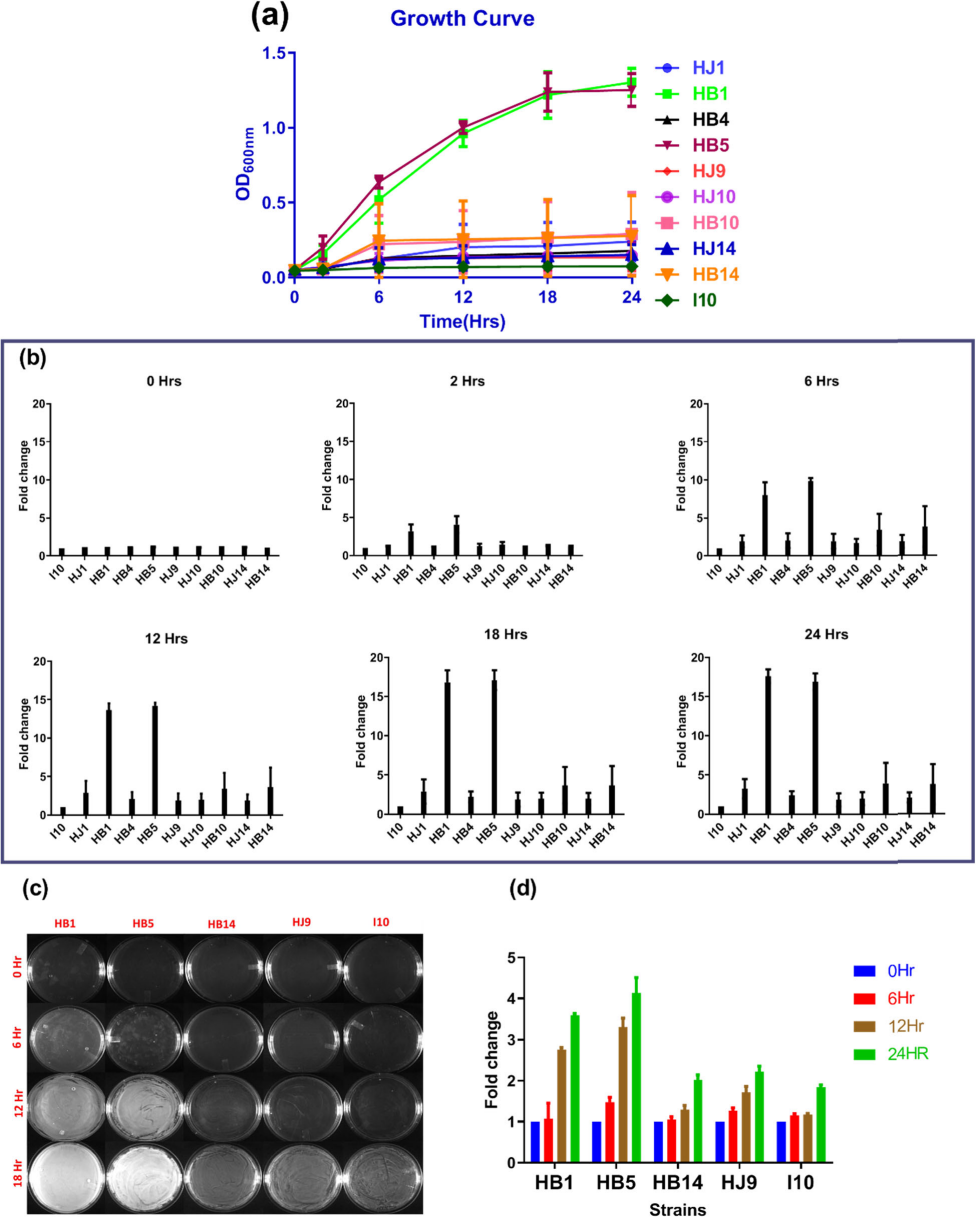
Growth pattern of isolated *H. pylori* strains. **a** The growth pattern of clinical isolates (HJ1, HB1, HB4, HB5, HJ9, HJ10, HB10, HJ14, HB14) and reference strain (I10) under specific microaerophilic condition assessed at 0, 6, 12, 18 and 24 h. The data are the mean ± SD (*n* = 4) of two independent experiments with technical replicate. **b**: Graphs are plotted for relative growth in comparison to I10 at 0, 2, 6, 12, 18, and 24 h. **c** Plate densitometry image of selected *H. pylori* isolates HB1 (lane first); HB5 (lane second); HB14 (lane third); HJ9 (lane fourth); and I10 (lane fifth) are showing growth till 24 h. The experiment is performed in duplicates, and the representative images are shown. **d** Fold change was calculated in comparison to 0 Hr

The growth curve of confirmed strains of *H. pylori* was determined by recording OD at 600 nm at various time points (0, 2, 6, 12, 18, and 24 h) and a curve was plotted (Fig. 2 a). Our results revealed that the growth of two clinical strains, namely HB1 and HB5 was significantly faster (*p* < 0.05) compared to the other seven clinical and one reference strain (I10). Interestingly, HB1 and HB5 have similar while not identical growth patterns at all examined time points (Fig. 2a). It is also fascinating that the growth pattern of HB1 and HJ1 was quite different, even though they were isolated from the same patient (Fig. 2a) Other clinical strains (HB4, HJ9, HJ10, HB10, HJ14, and HB14) shows a similar growth pattern as I10 until 24 h.

To better understand the growth pattern of isolated *H. pylori, g*raphs were plotted in the form of a bar chart at all recorded time points (0, 2, 6, 12, 18, and 24 h) and were compared with the reference strain (I10) (Fig. 2b). Three to five folds faster growth was observed in HB1 and HB5 compared to I10 at 2 h, and further, it increased up to 24 h (Fig. 2b). Moreover, isolates such as HJ1, HB10, and HB14 were showing moderately higher growth (3 to 5 folds) compared to reference strain I10 from 6 h onwards (Fig. 2b). Importantly, the growth of HB1 and HB5 was steadily increasing up to 18 h. Hence, our study reflected two fast-growing strains (HB1 and HB5) compared to other clinical isolates HJ1, HB4, HJ9, HJ10, HB10, HB14, HJ14, and reference strain I10 (Fig. 2b).

### Confirmation of active component of oral rinses through LC-MS

The active components in the oral rinses of A, B, C, D, and E was reconfirmed through LCMS at the Sophisticated Instrument Centre facility at IIT Indore (Supp. Fig. 2). The results validate the presence of labeled active components in them. We have got exact mass spectra at 304.5, 253, 205.1, 212.1, and 102.12 for CPC, chlorhexidine, clove oil, thymol, and povidone/iodine respectively (Supp. Fig. 2). Further, to evaluate the efficacy of selected oral rinses *H. pylori* growth analysis after treatment was performed.

### The growth pattern of selected clinical isolates of *H. pylori* after treatment with oral rinses

**Fig. 3:**
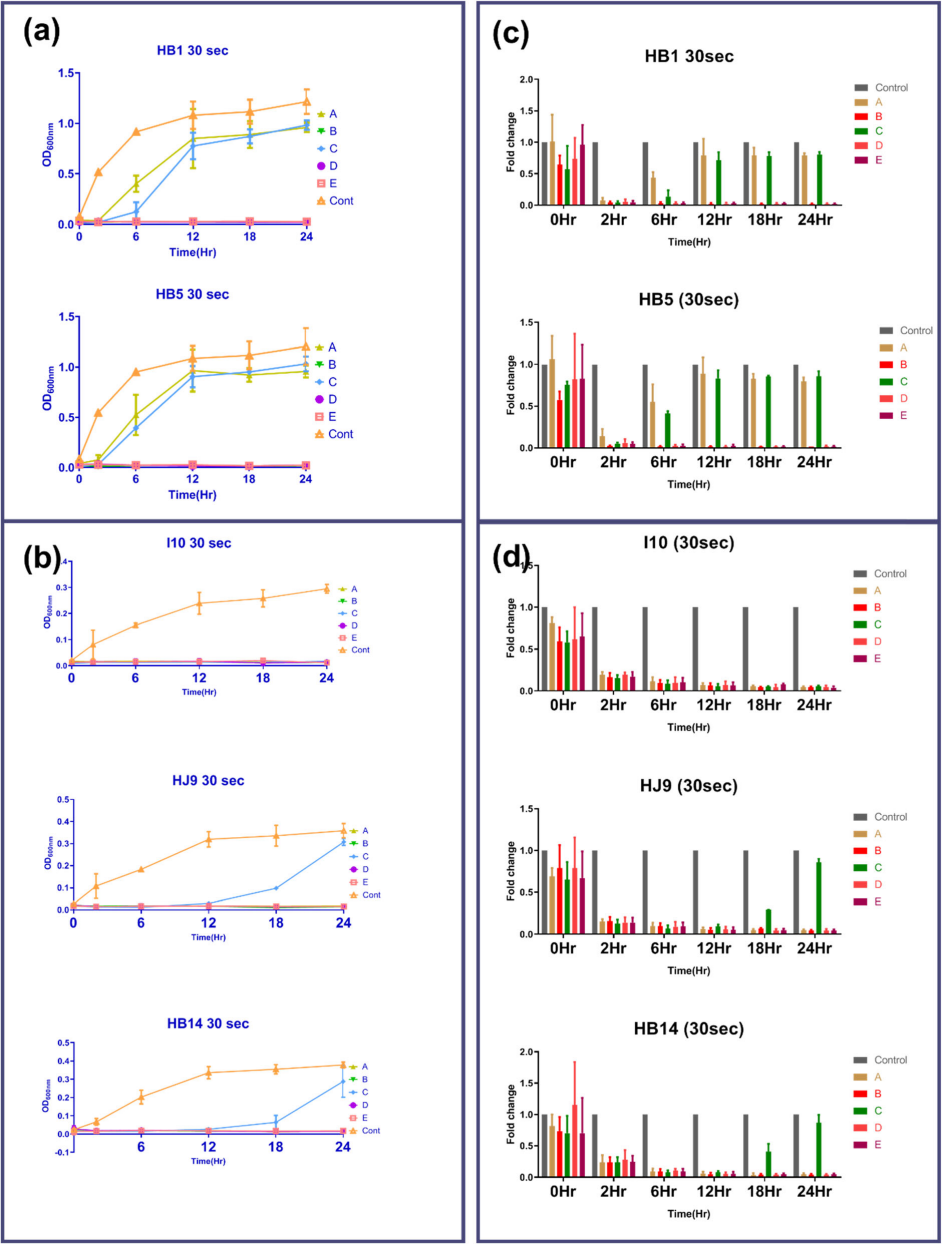
Growth pattern of *H. pylori* isolates after treatment with oral rinses for 30 s. Treatment of oral rinses (A, B, C, D, and E) was given to 6X10^7^ of *H. pylori* for 30 s, and growth was observed until 24 h compared to untreated control. Graphs reflect the growth of (**a**) fast (HB1 and HB5) and (**b**) slow-growing (HJ9, HB14, and I10) isolates. Relative growth of (**c**) fast (HB1 and HB5) (**d**) slow-growing (I10, HJ9, and HB14) was estimated compared to untreated control. The data are the mean ± SD of two independent experiments with technical replicate (*n* = 4, mean ± SD)

All chosen oral solutions recommend 30 s oral rinsing for effective plaque control. Further, our two fast-growing (HB1 and HB5), two slow-growing (HJ9 and HB14), and a reference strain (I10) were selected for this experiment (Fig. 3a, b, c, d). Interestingly, we observed that oral rinses A and C were not able to stop the growth of fast-growing strain after 30 s treatment (Fig. 3a). Although, growth of fast-growing strain was again increased after 2 h post-treatment with solution A and C while solution B, D, and E were able to inhibit the growth until 24 h post-treatment. We have found that solution A was least efficient in the control of HB1 growth followed by C. The substantial growth of HB1 was observed from 6 h onwards when treated with solution A and 12 h post-treatment when treated with oral rinse C (Fig. 3c). Moreover, the growth of another fast-growing strain, HB5, treated with oral rinses A and C, was suppressed until only 2 h (Fig. 3c). There was considerable growth of HB5; 6 h onwards with A, and C treatment. Importantly, oral rinses B, D, and E were able to suppress the growth of HB1 and HB5 until 24 h in this study (Fig. 3a, c). Additionally, slow-growing strain such as HJ9 and HB14 were not able to grow until 12 h with all used oral solutions (Fig. 3b). However, these strains start growing from 12 h onwards when treated with solution C (Fig. 3b). Notably, our reference strain, I10, has not shown any growth after treatment with all oral rinses until 24 h (Fig. 3b, d). In all these experiments, we have used untreated strains as positive controls and culture media as a negative control. A combination of effective oral rinses was used to evaluate their efficacy on treatment for a shorter duration.

### Selective oral rinse are restricting *H. pylori* growth even at shorter exposure

**Fig. 4:**
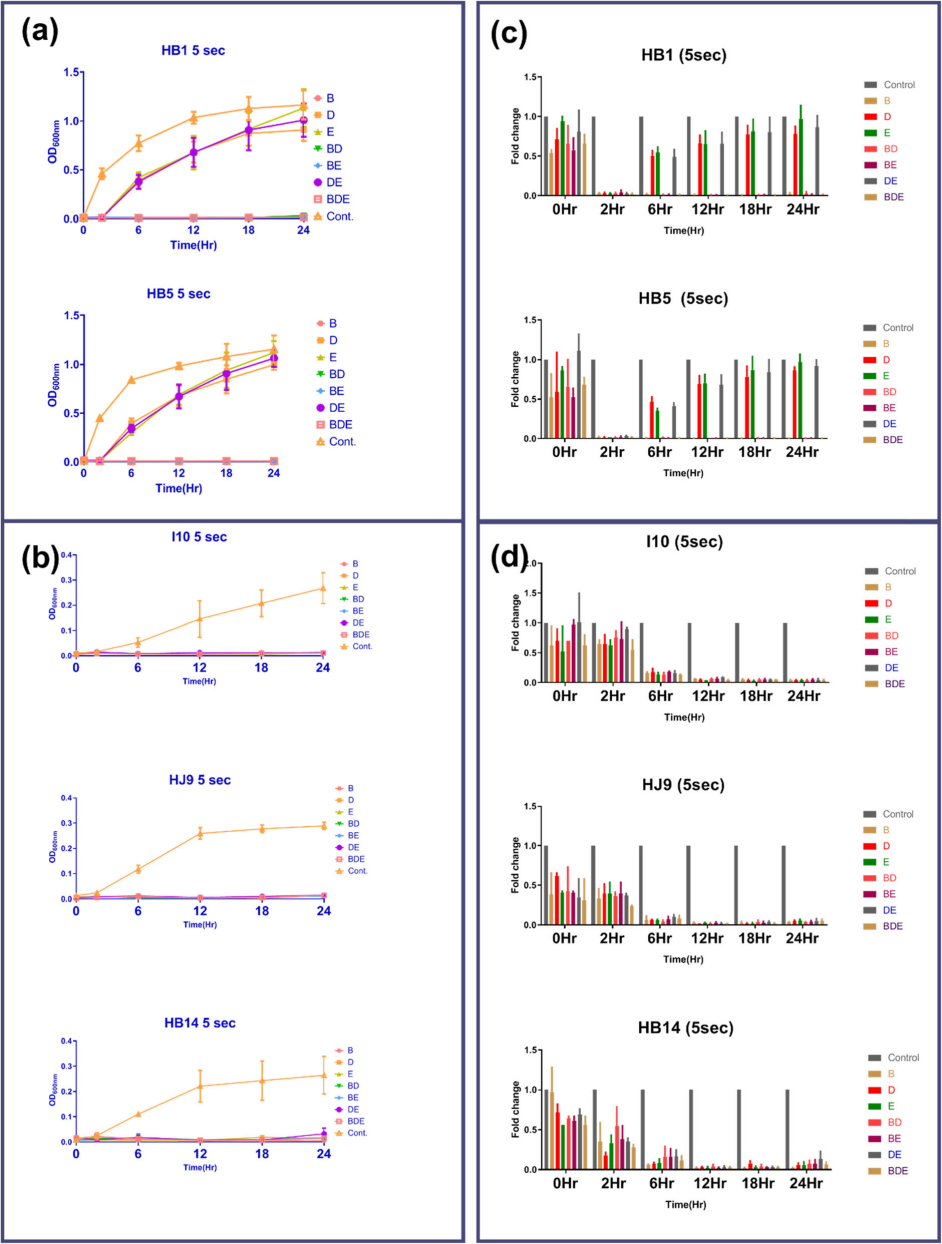
Treatment with selected oral rinses for a shorter duration. 6X10^7^ of *H. pylori* were treated with selected oral rinse alone and in combination (B, D, E, BD, BE, DE, and BDE) for 5 s. Growth was observed until 24 h in comparison to untreated control. Growth curve of (**a**) fast-growing isolates (HB1 and HB5) and (**b**) slow-growing isolates (HJ9, HB14, and I10). Relative growth of fast HB1 and HB5 (**c**) and in of slow-growing I10, HJ9, and HB14 (**d**) compared to untreated control. The data are the mean ± SD of two independent experiments with technical replicate (*n =* 4, mean ± SD)

Oral rinse B, D, and E were found to be effective in controlling the growth of slow as well as fast-growing strains at 30 s treatment. Hence, we investigated the effect of these selected solutions, alone (B, D, and E) and in combination, (BD, BE, DE, and BDE) for a shorter duration of treatment (5 s) compared to recommended 30 s (Fig. 4a, b). Even the 5 s treatment to fast-growing *H. pylori* isolates with all efficacious oral rinses alone and in combination were able to restrict the growth until 2 h (Fig. 4a, c). Surprisingly, data recorded 6 h post-treatment were demonstrating the growth of HB1 and HB5 with D, E, and their combinations. Importantly, all groups in which solution B is included shows to be restricting the growth of HB1 and HB5 (Fig. 4a, c). Interestingly, the growth of slow-growing strains HJ9, HB14, and reference strain I10, completely abolished with all the above solution combinations till 24 h (Fig. 4b). Moreover, when we treated these oral rinses for 10 s alone and in combination, a similar pattern was established as with 30 s (Suppl. Fig. 1).

### The growth pattern of *H. pylori* after oral rinses treatment on BHI agar plate

**Fig. 5:**
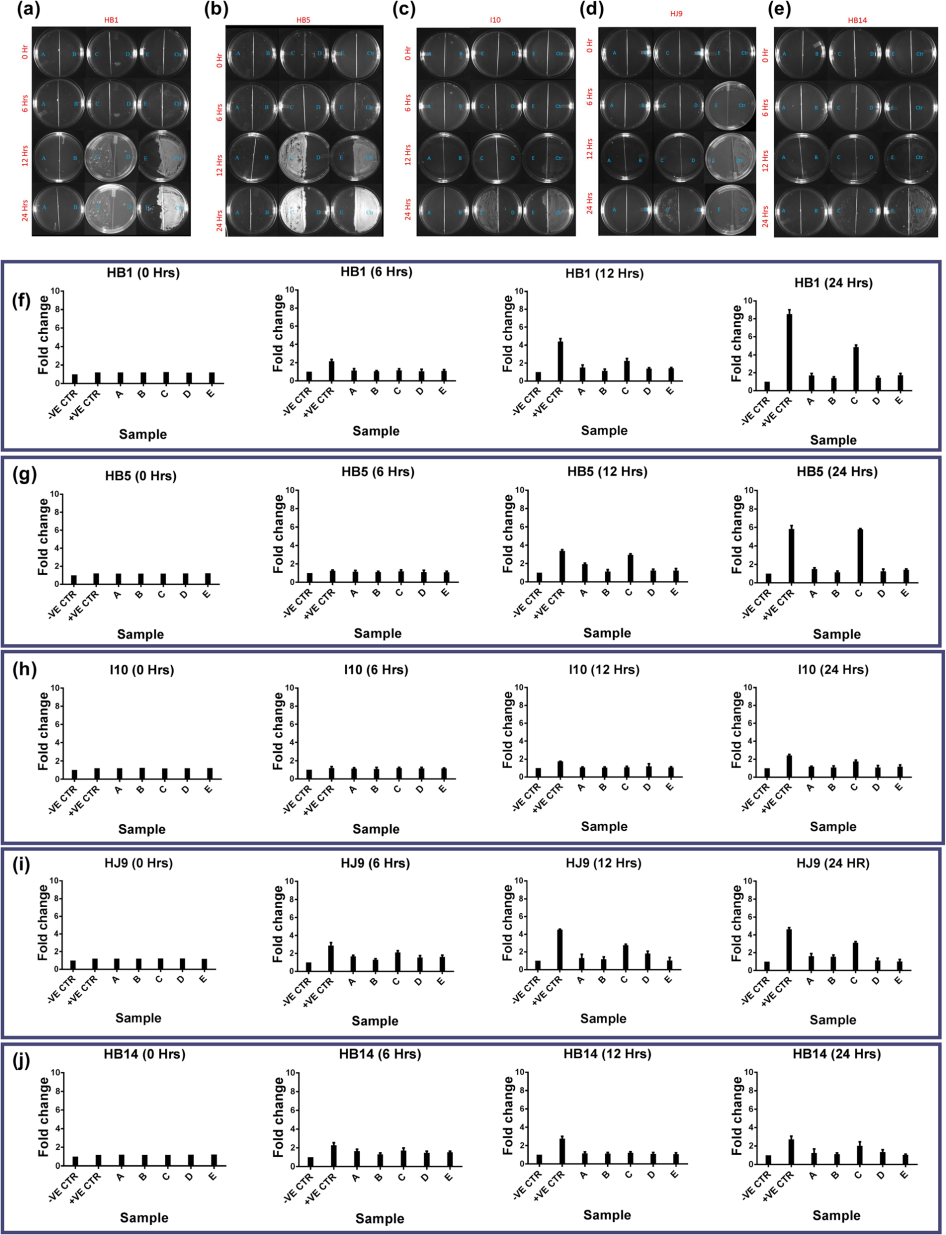
Plate densitometry of *H. pylori* after treatment with oral rinses*.* Oral rinses treatment of A, B, C, D, and E was given to 1X10^7^*H. pylori,* followed by spreading on half of the BHI Agar plate. Representative images showing growth of (**a**) HB1, (**b**) HB5, (**c**) I10 (**d**) HJ9 and (**e**) HB14 till 24 h Relative growth was estimated for fast-growing isolates (**f**) HB1 and (**g**) HB5; and slow-growing isolates (**h**) I10, (**i**) HJ9, and (**j**) HB14. Blank plates were considered as negative control and untreated isolates as a positive control. The data are the mean ± SD of two independent experiments with technical replicate (*n* = 4, mean ± SD)

In addition to solution treatment in liquid culture, we further evaluated the growth pattern of *H. pylori* isolates on a BHI agar plate after 30 s treatment with the selected oral rinse solution. Representative pictures of solution treated *H. pylori* strains are shown in Fig. 5a, b, c, d, and e. The images were quantified using Image J software (NIH), and graphs were plotted (Fig. 5f, g, h, i, j). As expected, solution treatment of C was ineffective, and *H. pylori* growth was observed in the case of fast-growing HB1 and HB5 after 12 h (Fig. 5a, b, f, g). Moreover, we also witnessed growth after 24 h for slow-growing strains (I10, HJ9, and HB14). Surprisingly, no growth was observed in the case of oral rinse A treatment in all the strains, contrary to the growth in liquid culture (Fig. 5). Again, as expected, no growth was detected after treatment with oral rinses B, D, and E at all the recorded time points. The solutions inhibit the *H. pylori* growth differentially; hence further, investigation of the known gastric cancer markers and *H. pylori* genes to assess the effect of oral rinses on its pathogenicity.

### Gene profiling of specific gastric cancer marker and *H. pylori* after oral rinse treatment

**Fig. 6:**
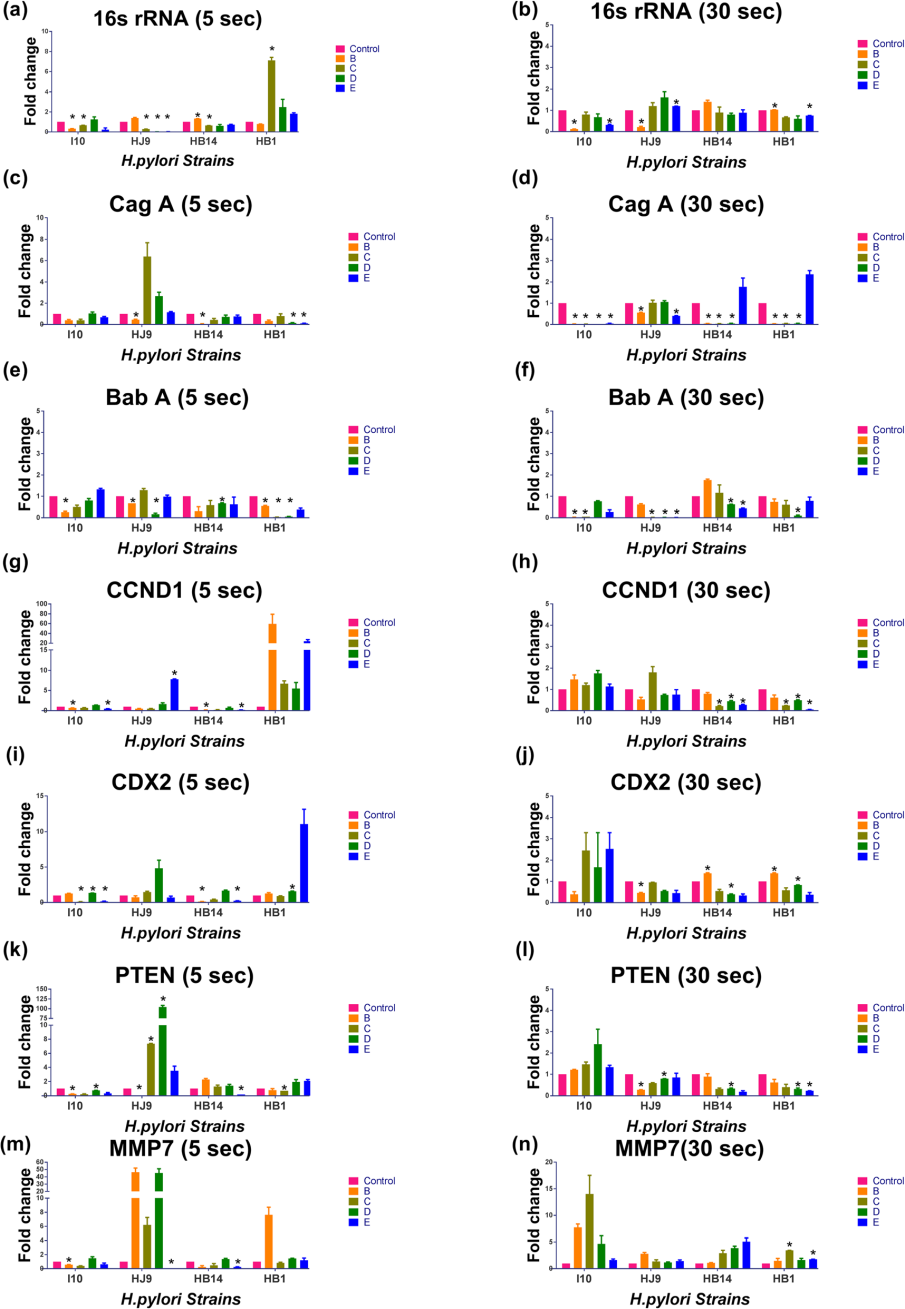
Investigation of *H. pylori* and gastric cancer genes: Treatment of solution was given to 6X10^7^ of *H. pylori* and incubated with 0.5X10^6^ AGS cell for 12 Hrs. RNA was isolated, and transcript level was determined by qRTPCR. Experiments were performed in duplicates. Expression of 16 s rRNA (**a**, **b**); CagA (**c**, **d**); BabA (**e**, **f**); CCND1 (**g**, **h**); CDX2 (**i**, **j**); PTEN (**k**, **l**); MMP7 (**m**, **n**) was evaluated on 5 and 30 s treatment respectively. AGS cells infected with wild type *H. pylori* treated as control in this study

Expression of *H. pylori* genes, namely 16 s rRNA, Cag A, and Bab A, were investigated in this experiment. Additionally, reported GC markers such as CCND1, CDX2, PTEN, and MMP7 were also included [27–30]. A mixed expression profile was observed on treatment with oral rinses (B, C, D, and E) for 5 s in the I10 strain. On treatment with solution B, *H. pylori* genes (16 s rRNA, CagA, and BabA) and GC markers (CCND1, PTEN, and MMP7) were down-regulated. However, expression was higher in CDX2 with 5 s treatment to *H. pylori,* followed by 12 h incubation with gastric epithelial cells. *H. pylori* genes, 16 s rRNA, and Cag A are down-regulated with the treatment of C and E (except HJ9) while Bab A was down-regulated with C (Fig. 6c, e). Moreover, PTEN and MMP7 were down-regulated with oral rinse solutions B, C, and E (Fig. 6k, m). Interestingly, 30 s treatment to I10 was able to abolish the expression of 16 s rRNA; CagA; and BabA with solution B; B, C, D, and E; and B, C, and E, respectively (Fig. 6b). Our results also revealed that expression of CDX2; and MMP7 were higher with solutions C, D, E; B, C, and D respectively with the 30 s treatment at 12 h time point (Fig. 6j, n). Similarly, when we treated another slow-growing strain HJ9 for 5 s with oral rinses followed by incubation with AGS. Strikingly a mixed response in gene expression profiling. In the case of PTEN; and MMP7, the expression is moderately enhanced with solutions C, D, and E; B, C, and D, respectively. Whereassolution B and E were able to diminish the expression of PTEN and MMP7, respectively (Fig. 6l, n). 30 s treatment of oral rinse B to the same strain followed by incubation shows slight downregulation in the expression of 16 s rRNA, CagA, BabA, CCND1, CDX2, and PTEN, however, the expression of MMP7 was an exception (Fig. 6). Further, when we treated HB14, another slow-growing strain, for 5 s with oral rinses solution followed by incubation with AGS, CagA, BabA, CCND1, CDX2, and MMP7 was considerably down-regulated with solution B (Fig. 6). Additionally, CCND1, CDX2, and MMP7 were minimally expressed with the treatment of B, C, and E, while PTEN is down-regulated with E (Fig. 6). Moreover, treatment of HB14 for 30 s and incubation with AGS, also leads to the downregulation of CCND1, CDX2, and PTEN with C, D, and E, while expression of MMP7 was unregulated with C, D, and E (Fig. 6n). However, CagA was abolished with B, C, and D (Fig. 6).

Furthermore, results reflect different gene expression profiles with the treatment of 5 and 30 s in fast-growing strain HB1 (Fig. 6). Expression of CagA; and BabA were down-regulated with the treatment of B, D, and E; and B, C, D, and E, respectively (Fig. 6c, e). Whereas the expression of CCND1; CDX2; and MMP7 were enhanced with B, C, D and E; E; and B, respectively (Fig. 6g, i, m). Treatment for 30 s to HB1 shows a minimal expression of CagA with solution B, C, and D, while; MMP7 was up-regulated with all the oral rinses (Fig. 6d, n). Induction of apoptosis in the cancer cell is one of the widely used treatment regimes against cancer [31]. Hence we have assessed apoptotic pathways that may be induced after growth inhibition of *H. pylori* due to the treatment of oral rinses.

### Status of apoptotic gene expression

**Fig. 7:**
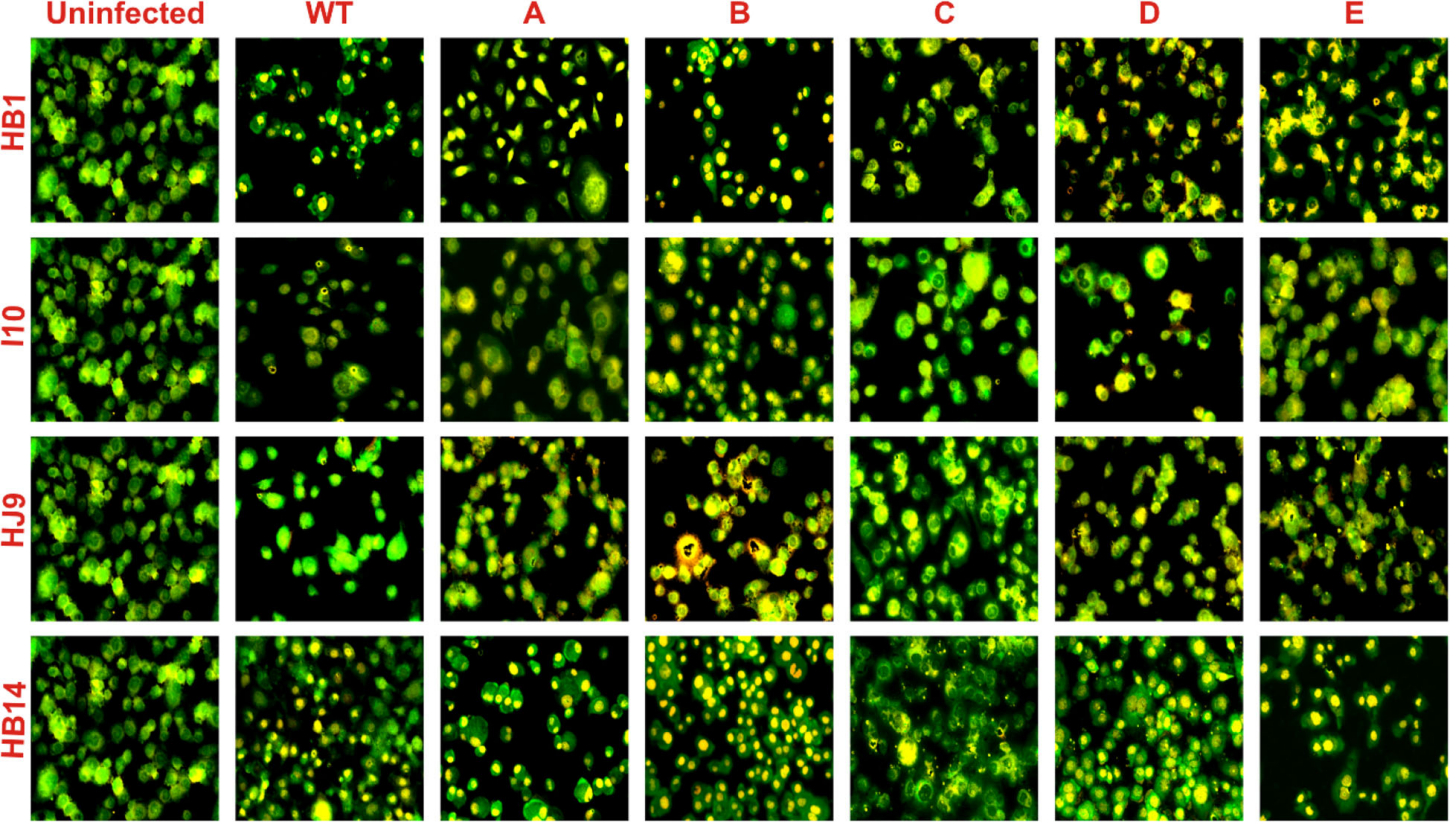
EB/AO dual staining assay. 0.25X10^6^ AGS cells were plated in 6 well plates and infected with 30 s oral rinse solution (**a**, **b**, **c**, **d**, and **e**) treated and wild type (WT) *H. pylori* isolates. Furthermore, 12 h post-infection, the cells were stained with EB/AO solution, and images were acquired. (**a**) HB1, (**b**) I10, (**c**) HJ9 and (**d**) HB14 infected and uninfected AGS cells

**Fig. 8:**
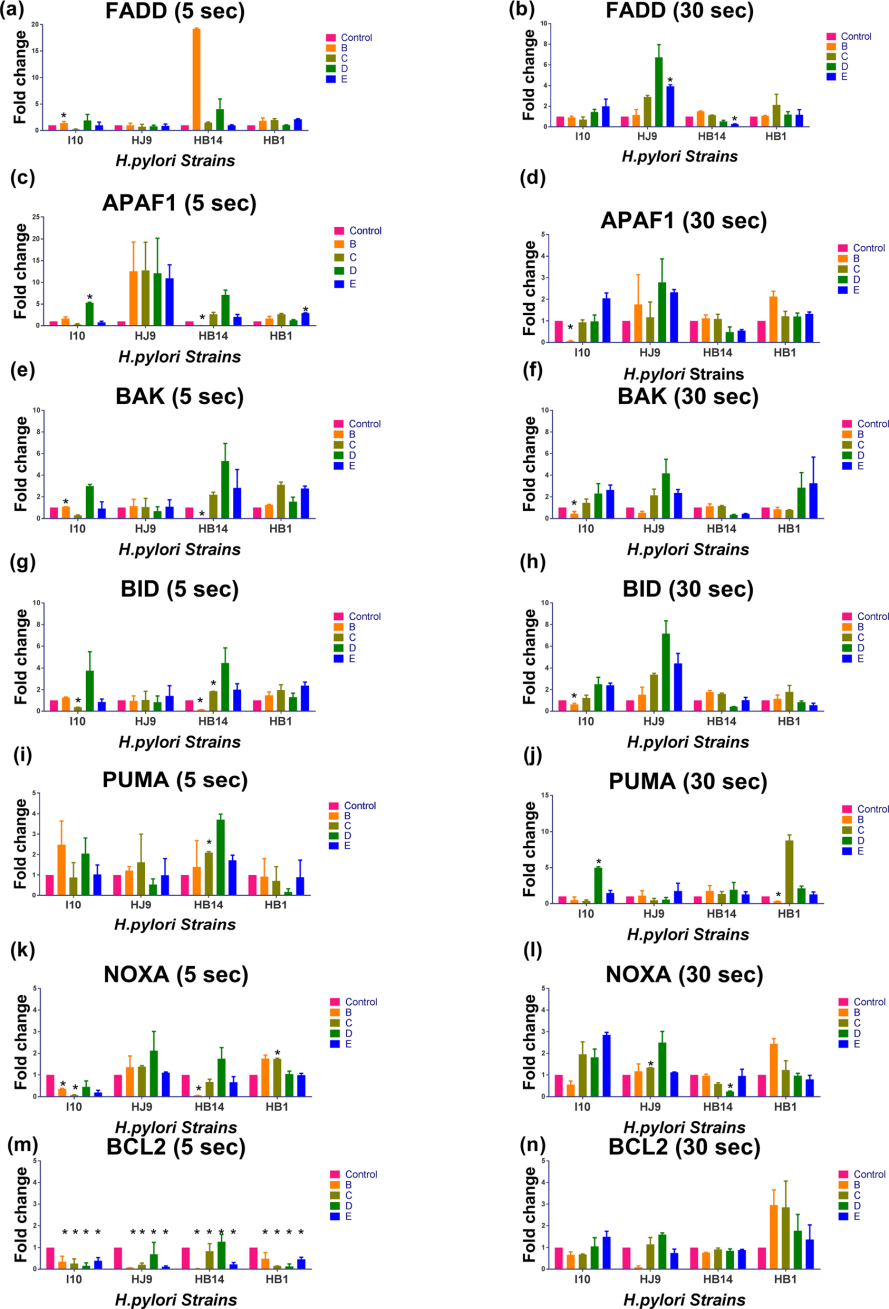
Status of apoptosis-related genes. Treatment of solution was given to 6X10^7^ of *H. pylori* and incubated with 0.5X10^6^ AGS cell for 12 Hrs. RNA was isolated, and the transcript level was determined by qRTPCR. Experiments were performed in duplicates. Expression of (**a**, **b**) FADD; (**c**, **d**) APAF1; (**e**, **f**) BAK; (**g**, **h**) BID; (**i**, **j**) PUMA; (**k**, **l**) NOXA; and (**m**, **n**) BCL2 was assessed after treatment for 5 and 30 s respectively. The data are the mean ± SEM of two independent experiments with technical replicate (*n =* 4)

Earlier studies have classified cells as live, apoptotic, and necrotic after EB/AO staining [32]. We investigated these cells (live, apoptotic, and necrotic) on infection with *H. pylori* treated with oral rinses for 30 s (Fig. 7). Additionally, the evaluation of apoptotic pathways, such as intrinsic/extrinsic/independent, was performed after treatment of *H. pylori* isolates with these solutions for 5 and 30 s through qRT-PCR (Fig. 8). 5 s exposure of solution D in I10 strain was able to enhance the expression of APAF1, BID, and BAK (Fig. 8c, e, g). Interestingly, I10 treated with solution C and incubated with AGS cells were able to suppress all studied apoptotic genes (Fig. 8). Whereas, other solutions were not able to change the expression patterns of these genes considerably. Furthermore, 30 s treatment of I10 and incubation with AGS cells show a different pattern (Fig. 8). I10 treated with solution B was able to reduce the expression of all the selected genes except FADD (Fig. 8). However, treatment with solution D and E were slightly enhancing the expression of all pro-apoptotic genes (Fig. 8).

Furthermore, when we applied these solutions for 5 s on HJ9 and incubated with AGS cells, an upsurge in APAF1 expression, while BCL2 found down-regulated except in solution D (Fig. 8c, m). Surprisingly, 30 s treatment of HJ9 reflected a varied gene expression compared to 5 s. Oral rinse C, D, and E with 30 s exposure were able to up-regulate all apoptotic genes except NOXA and PUMA (Fig. 8). However, the expression of anti-apoptotic BCL2 was reduced with oral rinse B (Fig. 8n).

Application of these oral rinses for 5 s on HB14 followed by incubation with AGS cells demonstrated that oral rinse D up-regulates all pro-apoptotic genes (Fig. 8). Interestingly, treatment with B was able to enhance extrinsic apoptotic regulator FADD and reduce the expression of all used intrinsic markers. It also diminished the expression of BCL2 (Fig. 8), fascinatingly, all apoptotic markers except PUMA were considerably down-regulated with the 30 s treatment of solution D (Fig. 8). Moreover, the expression of FADD, APAF1, and BAK, were also reduced with solution E (Fig. 8b, d, f).

Furthermore, in the case of fast-growing strain, HB1, solution C and E were slightly up-regulating the expression of FADD, APAF1, BID, and BAK (Fig. 8a, c, e). In addition to this, solution B treated cells were able to up-regulate FADD, APAF1, BID, and NOXA (Fig. 8a, c, g, k). Treatment of solution C for 30 s to the same strain showed up-regulation of FADD, BID, and PUMA (Fig. 8b, h, j). In contrary to this, slightly up-regulation was observed for BCL2 in all used solutions.

## Discussion

Various studies suggest an association between *H. pylori* and GC [1]. Infection of *H. pylori* in less developed Asian countries like India, Pakistan, Thailand, and Bangladesh is more prevalent than in more developed Asian countries like Japan and China. Interestingly, the occurrence of GC is lower in these less-developed Asian countries compared to Japan and China [13]. A similar enigma has been reported from Africa as compared to the Western countries [33]. The prevalence of *H. pylori* infection and the occurrence of GC may not appear to be proportionate around the world [9]. This may be attributed to the variations in the *H. pylori* strain pathogenicity and other associated risk factors for GC. It is essential to understand the discrepancy in *H. pylori* pathogenicity due to geographical and host anatomical locations.

In the present study, we were able to demonstrate significantly different growth patterns of isolated *H. pylori* collected from gastric biopsy and juice samples. These findings motivated us to validate the growth pattern of various clinical *H. pylori* isolates through plate densitometry. The *H. pylori* isolation from two different niches of the same person may also lead to the differential growth pattern. For the first time, our study revealed that *H. pylori* isolated from the biopsy grow aggressively compared to those isolated from gastric juice of the same subject. For example, HJ1 and HB1; HJ10 and HB10; and HJ14 and HB14 were isolated from two different niches of the same subjects. There was a significant difference in the growth pattern of HJ1 and HB1, which was not much remarkable in the case of HJ10 and HB10, as well as HJ14 and HB14. This finding demonstrates the importance of infection sites and their micro-environment for *H. pylori* dependent disease progression. Similar findings have been reported in pneumococcal infection [34]. Hence, these results show a specific pattern for the adaptation of *H. pylori,* which may act as a milestone in disease progression and subjected to further investigation.

Earlier, Marshall et al. showed that *H. pylori* eradication resulted in rapid gastric ulcer healing (92% vs. 61%) and lower relapse rate (21% vs. 84%) than non-eradication [35]. Importantly, resistance to antibiotic treatment is one of the major causes for the development of primary *H. pylori* infection to chronic gastritis and GC [36]. Studies reported that *H. pylori* isolated from the Indian population are resistant to its specific antibiotics [37]. Additionally, some strains of *H. pylori* are also resistant to triple therapy, which further increases the complication in the eradication procedure [38].

The transmission mode of *H. pylori* is an oral-oral or oro-fecal route [39]. Therefore, the oral cavity may serve as a reservoir for *H. pylori* [16]. Earlier studies demonstrated that relapse of *H. pylori* infection is mainly due to the presence of its extra-gastric reservoir in the oral compartment [40]. Hence, eradication of *H. pylori* in the mouth may help in restricting its transmission and relapse from mouth to stomach [21]. In our study, the approach is to eliminate *H. pylori* in the oral microenvironment using mouth rinses. It will further help in treating the infection and will enhance the treatment outcome in gastric abnormalities.

Various antiplaque and antimicrobial agents are known for inhibiting the growth or killing the target bacteria present in the oral microenvironment. These solutions may consist of chlorhexidine [41], essential oil [42], cetylpyridinium chloride [43], povidone-iodine [44], etc. In our study, we investigated the effect of these oral rinses on *H. pylori* growth and its carcinogenic abilities.

Based on differential growth observation of these isolates, we selected two fast (HB1 and HB5) and two slow-growing strains (HJ9 and HB14), along with one reference strain I10, for further study. Oral rinses B, D, and E, were efficient in killing fast-growing strains with 30 s incubation. In contrary to this, oral rinse A and C act as bacteriostatic and inhibit the growth until 2 h. Additionally, in the case of slow-growing strains (HJ9 and HB14), all used solutions except C are useful, while, solution C act as bacteriostatic up to 12 h. Interestingly, all used oral rinses were found to be efficient in killing reference strain I10, which has been grown for a long time in laboratory conditions. In order to understand the pathogenic islands of various clinical isolates, there is a need for further investigation of differential oral rinse response in clinical isolate vs. reference strain even though their growth patterns are similar.

To further validate the above experiment, plate densitometry was performed. It was observed that the growth of fast-growing strains (HB1 and HB5) with the treatment of solution C was detected at 12 h in comparison to 6 h of untreated. However, in liquid culture growth was observed at 6 h compared to 2 h in untreated. The growth of isolated strains (HJ1, HB4, HJ9, HB10, HJ10, HB14, and HJ14) was similar to the reference strain I10, whereas, HB1 and HB5 show about 15 fold higher growth after 12 h. These fast-growing strains reflect higher growth at 2 and 6 h by 4 and 9 folds, respectively.

Importantly, the growth of HB1 and HB5, which was observed with solution A treatment in liquid culture, was not visible in the plate densitometry. Moreover, the growth of wild type fast-growing strains could be detected within 2 h in liquid culture compared to 6 h in plate densitometry.

The use of oral rinse is a common practice, while prolonged and repetitive use of these oral rinses has adverse effects on the users [45, 46]. A study conducted by McGaw & Belch shows negligible toxicity associated with the use of chlorhexidine mouth rinses through radiolabeling methods [47]. However, such studies were conducted in the 1980s; hence, further investigations with modern detection techniques are needed in long term follow-up. Research on povidone-iodine, reveals that it can cause acute renal failure, mainly when absorbed through mucosal surfaces [48, 49]. Even diluted solutions of povidone-iodine (0.1 to 20%) are toxic to human fibroblasts, granulocytes, and monocytes [50]. Toxicity of another solution, cetyl pyridinium chloride, is noticed with vomiting, diarrhea, and abdominal pain. Ingestion of this solution in concentrated form may produce burns of the mouth, pharynx, and esophagus [51]. Additionally, hemorrhagic GI tract necrosis and peritonitis have also been reported [51]. Prolonged exposure of common constituents of oral rinses like essential oils, menthol, thymol can act as potential allergens in various ethnical races [52]. Moreover, some manufacturers produce alcohol-based mouthwashes, which can cause complications like irritation of oral mucosa and may be hazardous if ingested accidentally during pregnancy [53].

One of our focus in this study was to reduce the time of exposure to minimize the possibility of toxicity caused by these solutions. To achieve our goal, we performed the 5 s treatment and compared it with 30 s treatment of *H. pylori* isolates. We selected those oral rinses which were efficient in the 30 s treatment in combinations and alone. As expected, the growth of slow-growing strains (I10, HJ9, and HB14) was controlled through all used oral rinses at 5 s incubation. However, only solution B and its combination seem to be potent for all *H. pylori* strains. Strikingly, oral rinses D and E, which were potent on 30 s treatment, have not shown comparable results with fast-growing strains. The high efficacy of solution B may be attributed to its sizeable dicationic molecule (Chlorhexidine), which can adsorb onto negatively charged bacterial cell walls [54]. This increases the permeability of the inner membrane and leads to the leakage of low molecular weight components. At 0.2% concentration, this damage is permanent and hence acts as a bactericidal agent [54].

The Cag pathogenicity island (cag-PAI) is one of the major virulence determinants of *H. pylori*. Irrespective of the growth pattern variation in isolated *H. pylori* strains, a significant downregulation of the CagA gene was observed with solution B. Also, prolonged exposure to solution C and D to I10, HB14, and HB1 diminish the expression of CagA. Here Cag A expression imitates the growth of *H. pylori* after solution B treatment.

EB/AO staining has been used as a gold standard to differentiate between live, apoptotic (early/late), and necrotic cells. We observed that *H. pylori* treated with solution B and incubated with AGS shows more cells in the late apoptotic stage. While with solution C, D, and E treatment, cells are either in the early apoptotic stage or live. This reflects that solution B is most effective among all the studied oral rinses. To understand the growth arrest of *H. pylori* through used oral rinses, we have mapped several mechanisms of cell death, mainly apoptosis and necrosis. Interestingly, extrinsic apoptotic marker FADD is slightly enhanced in slow-growing strain HJ9 and HB14 with selective oral rinses. Although, studies suggest that FADD is expressed on the surface of cytotoxic T lymphocytes (CTLs) and natural killer (NK) cells as part of their armamentarium against infected or transformed cells [55].

Moreover, markers for the intrinsic pathway, APAF1 show higher expression during shorter exposure of solution B, C, D, and E to HJ9, while the result is not similar for more prolonged exposure. Notably, BID shows selective up-regulation with solution D in slow-growing strains. Studies have demonstrated that both APAF1 and BID belongs to the BCL2 family and act with the mitochondria-related apoptotic pathway [56, 57]. However, expression pattern for BAK, PUMA, and NOXA was not decisive with used oral rinses s for all included *H. pylori* strains. PUMA and NOXA belong to the pro-apoptotic BH3 only family, which regulates BCL2 activity [58]. Interestingly, the anti-apoptotic gene, BCL2, was noticeably down-regulated in 5 s treatment while not in 30 s with the selected oral rinses.

## Conclusion

*H. pylori* adaptation to different physiological habitat in the host may be responsible for the differences in its growth and pathogenicity. For avoiding the challenges of relapse and antibiotic resistance, we used oral rinses and found that these are effective against CagA expression in *H. pylori*. Some of the variability in outcome can be attributed to different bacterial strain specificity, host susceptibility, and the type of response elicited in the infected host. There is a need for a detailed study about the molecular pathways modulated by the oral rinses in bacteria and surrounded host cells. These studies will also open a broad scope to apply various bactericidal combinations for the treatment and eradication of *H. pylori* infection.

## Methods

### Patient recruitment

The endoscopic procedure was performed after getting informed written consent from the patients. The protocol for the present study was approved by the ethical committee of the Indian Institute of Technology Indore, as well as Choithram Hospital Indore, and all procedures were performed by following the revised declaration of Helsinki 2000. Before collection of the sample written consent of the participants were obtained in a consent form. We collected only Rapid Urease Test (RUT) positive 14 biopsies (male = 9 and female = 5) and 11 gastric juice (male = 6, female = 5) samples from gastritis patients (Table 1). Patients undergoing antibiotic treatment against *H. pylori* were excluded for sampling. For further processing, biopsy samples were immediately placed in a microcentrifuge tube containing Brucella broth (BD Difco Cat No. 8806541) with 20% glucose (Hi-media, Cat No. TC130), while gastric juice was collected in sterile 15 ml centrifuge tube. Samples were transported to IIT Indore in ice.

### Culturing of *H. pylori* from clinical samples

The biopsy samples were homogenized by using a glass rod. One loopful of the homogenous sample was streaked on Columbia agar plate (Himedia ME144) containing the *H. pylori* selective antibiotics, (5 mg/L cefsulodin, 10 mg/L vancomycin, 5 mg/L amphotericin B, 5 mg/L trimethoprim and 10% defibrinated blood, BD, Cat. No. 254005). The plates were incubated in a microaerophilic chamber (Whitley DG 250) containing specific growth conditions (i.e., 85% N_2_, 10% CO_2_ and 5% O_2_) at 37 °C. The same procedure was followed for gastric juice samples, and the colonial growth was observed for the next 3–4 days. *H. pylori* isolated form biopsy and gastric juice samples were named as HB and HJ, respectively, followed by a number representing the sequence of sampling. In the present study, we also used I10 as a reference *H. pylori* strain, which was kindly gifted by Dr. Ashish Kumar Mukhopadhyay from the National Institute of Cholera and Enteric Diseases (NICED) Kolkata.

### Culture of clinical isolates in liquid and solid growth medium

A single colony was picked from the Columbia agar plate of each sample and inoculated in brain heart infusion media (BHI, Cat. No. 237500- BD Brain Heart Infusion broth), containing 10% Fetal Bovine Serum (FBS Hi-media, Cat. No. RM-10432) with 3X *H. pylori* selective antibiotics in a snap cap tube (BD, Cat. No. 352001) [59]. Simultaneously, it was also streaked on a BHI agar plate containing the same concentration of FBS and antibiotics. Both broth and plate were incubated in a similar growth condition, as described above.

### Gram staining

In order to identify isolated strains, Gram staining was performed. Preparation of smear was done after suspending a new culture in 100 μl PBS followed by air-drying and heat-fixation over the flame. The smear was flooded with crystal violet for 60 s, and the excess stain was washed off with distilled water. The smear was again flooded with Gram iodine for 60 s, followed by destaining with 95% ethanol. The slides were further rinsed with distilled water and blot dried, followed by counterstaining with safranin for 30 s. The slides were again washed and observed under the microscope (100X - oil immersion lens) (Nikon Eclipse E100 upright microscope) (Fig. 1).

### Growth curve

The isolated *H. pylori* strains were analyzed for the growth pattern until 24 h. In brief, the isolates were cultured in a 14 ml round bottom snap cap tube (BD) in biological duplicates by setting initial OD_600_ 0.05, which correspond to approximately 80 million CFU per ml [59]. Further, they were incubated in the microaerophilic chamber, as mentioned above. 150 μl grown culture was placed in duplicate in 96 well flat-bottom plates, and OD was recorded at 600 nm (Synergy H1 Hybrid Multi-Mode Reader, BioTek). The final OD value was normalized with media as a negative control.

### DNA isolation

*H. pylori* culture was harvested in phosphate-buffered saline for DNA extraction at OD_600nm_ of 0.2–0.6. The pelleted cells were suspended in extraction solution (10 mM Tris pH 8.0, 15 mM NaCl, 10 mM EDTA, 0.5% SDS) and kept at 55 °C for 1 h. Proteinase K (Thermo fisher scientific) solution (20 mg/ml) was added (1 mg/ml), and samples were incubated overnight at 37 °C. RNAse-A (Himedia) was added (0.1 mg/ ml) to the solution and kept at 37 °C for 1 h. The DNA was then extracted with the phenol-chloroform-isoamyl-alcohol method, as reported previously [60].

### PCR detection

*H. pylori* DNA samples were amplified by Platinum™ Taq DNA Polymerase (Invitrogen™, Cat. No.10966026). The reaction volume for PCR was 50 μL (50 mM KCl, 1.5 mM MgCl_2_, 200 mM dNTPs, 10 mM Tris-HCl (pH = 8.3), 10 pmol primer, 2.5 units Taq polymerase and 100 ng of DNA template). 16 s rRNA specific forward primer 5′ CTGGAGAGACTAAGCCCTCC 3′ and reverse primer 5′ ATTACTGACGCTGATTGCGC 3′ respectively were used for amplification (product size − 110 bp). The amplification was carried out with initial denaturation at 95 °C for 7 min, followed by 40 cycles of denaturation, annealing, extension, and a final extension at 94 °C for 2 min, 55 °C for 30 s, 72 °C for 30 s and 72 °C for 10 min respectively. Analysis of the amplified products was done by gel electrophoresis using 2.5% agarose gel and stained with 0.5 μg/ml ethidium bromide. Product size was confirmed by using a 50-bp DNA ladder (Hi-Media, cat no. MBT130). The image of gel was acquired on a gel documentation system (ImageQuant LAS 4000, GE Healthcare Life Sciences).

### Oral rinses

Five different commercially available mouthwash solutions were purchased from local pharmacy store and were assigned name A, B, C, D & E. Mouthwash A contains cetylpyridinium chloride 0.075% w/w, mouthwash B contains chlorhexidine (0.2% w/v), mouthwash C contains naturally derived clove oil (0.1 mg/gm) (cloves extracts), mouthwash D contains thymol (thyme) 0.064%, methyl salicylate (wintergreen) 0.06% and eucalyptol (eucalyptus) 0.092%, and mouthwash E contains 2% w/v povidone-iodine.

### Confirmation of the active component of oral rinses through LC-MS

Mass and spectral analysis were done by Bruker Daltonik, Benchtop, High-Performance Electrospray Ionization Quadrupole time-of-flight LC-MS spectrometer designed for estimation of an exact mass of the components present in mouthwash solutions. The bactericidal function of cetyl-pyridinium chloride (CPC) or 1-hexadecyl pyridinium chloride or chlorhexidine gluconate (CHG), clove oil, menthol/thymol, and povidone/iodine is well known and used in various antibacterial products like mouthwashes, throat sprays, nasal sprays.

### *H. pylori* growth inhibition by oral rinses

Povidone-iodine (2%) was diluted in water (1:1), while other mouthwashes were used in provided concentrations. A fixed number of *H. pylori* (6 × 10^7^) were incubated with 1 ml of all mouthwashes for 30 s, 10 s, and 5 s, followed by centrifugation at 3000 rpm for 5 min. The control group was left untreated. Pellets were suspended in 0.5 ml of BHI media containing 10% FBS and selective antibiotics followed by incubation in microaerophilic conditions, as mentioned above. 150 μl of culture was taken in each well in duplicates in a flat bottom 96 well plate, and optical density was recorded at 600 nm (Synergy H1 microplate reader, Biotek) at 0, 6, 12, 18, and 24 h time points, and growth curve was plotted.

### Densitometry of *H. pylori* growth on BHI agar plate

A plate densitometry study on a BHI agar plate was performed to validate the growth pattern in the liquid medium. In order to check the growth of *H. pylori* (HB1, HB5, HJ9, HB14, and I10) on BHI agar plate, 1X10^7^ bacteria were taken (OD_600_ 0.3 represents 500 million CFU per ml) [59] and suspended in 100 μL of BHI broth and then spread by using glass spreader. Further, images of plates were obtained at 0, 6, 12, and 24 h, and data were analyzed by measuring the mean grey value using Image J software (NIH) (Fig. 2c). In addition to this, the growth of bacteria on a BHI agar plate after 30 s solution treatment was also determined. 0.5X10^7^ bacteria were taken and subjected to centrifugation at 3000 rpm for 5 min. The supernatant was discarded, and the pellet was treated with 333 μL of the selected oral rinses (A, B, C, D, and E) for 30 s. The treatment was stopped by centrifugation at 3000 rpm for 5 min, and the supernatant was discarded. The pellets were suspended in 50 μL of BHI broth and spread in half of the plate and incubated in a microaerophilic condition followed by taking an image at various time intervals (Fig. 5). We have shown representative images of *H. pylori* growth on BHI plates. Further, for better understanding, we calculated the fold change in growth, and the obtained data were plotted into graph Fig. 2(c, d) and Fig. 5.

### RNA isolation and gene expression study through qRT-PCR

Fixed number (6X10^7^ CFU per ml) of *H. pylori* isolates (I10, HJ9, HB14, HB1) were treated with 1 ml of oral rinse for 30 s and 5 s. Here, oral rinse A was excluded from cell culture study because of alcoholic constituents. Further, *H. pylori* isolates were incubated with AGS cells under specific conditions (5% CO_2_, 37 °C) for 12 h. At 12 h time point, the pellet was collected by centrifugation (1600 rpm for 5 min) and washed twice with PBS. Total RNA was isolated by using Ribozol reagent (VWR™ Cat No. N580) as per the manufacturer’s instruction. cDNA was synthesized using the PrimeScript 1st strand cDNA Synthesis Kit (Takara, Cat No. RR820Q) according to the manufacturer’s instruction. Quantitative real-time PCR (qRT-PCR) analysis was performed using the AriaMx Real-Time PCR System (Agilent technologies 5301 Stevens Creek Blvd Santa Clara, CA 95051 USA), for assessment of gastric cancer marker genes (CCND1, CDX2, PTEN, and MMP7), apoptotic genes (FADD, APAF1, BID, BAK, NOXA, PUMA and BCL2) and pathogen-associated genes (CagA, BabA, and 16 s-rRNA), (Supp. Table 1).

### Ethidium bromide and acridine orange (EB/AO) assay

EB/AO dual staining was performed for the assessment of the apoptotic, necrotic, and live-cells after infection with oral rinses treated *H. pylori*. The experiment was performed in duplicates, and the image was acquired by confocal microscopy (Olympus IX83) at 10X with 3X zoom in triplicates. The working concentration of acridine orange and ethidium bromide was 100 μg/ml each [61].

## Supplementary information

**Additional file 1: Figure S1.** Growth pattern of *H. pylori* isolates after treatment of solutions for 10 sec. **Figure S2.** Confirmation of chemical plaque control agents through LCMS **Table S1.** Genes specific primers included in this study.

## Data Availability

All-important data are presented in the manuscript or supplementary figures. Some other supporting information that may not be crucial or affecting result interpretation is not included. Moreover, these data can be available from the corresponding author on a reasonable request.

## Abbreviations

AlpA: Adhesins associated protein A
BabA: Blood group antigen binding adhesin A
BAK: Bcl-2 homologous antagonist/killer
BCL2: Apoptosis regulator Bcl-2
BID: BH3-interacting domain death agonist
CagA: Cytotoxin-associated gene A
CCND1: Cyclin D1
CDX2: Caudal type homeobox 2
CFU: Colony Forming Unit
FADD: FAS-associated death domain
HB: Human Gastric Biopsy
HJ: Human Gastric Juice
Hop: Hope family outer protein
MMP7: Matrix Metallopeptidase 7
NOXA: Phorbol-12-myristate-13-acetate-induced protein 1
OD: Optical Density
OiP: Outer membrane associated protein
PTEN: Phosphatidylinositol 3,4,5-trisphosphate 3-phosphatase and dual-specificity protein phosphatase
PUMA: Bcl-2-binding component 3
SabA: Sialic acid–binding adhesion
