# Supplementary material for: Oral rinses in growth inhibition and treatment of Helicobacter pylori infection
Source: BMC Microbiol. 2020 Mar 4;20:45. doi: 10.1186/s12866-020-01728-4 (PMC7055109; doi:10.1186/s12866-020-01728-4)
Supplement: Supplementary file 1 — Additional file 1: Figure S1. Growth pattern of H. pylori isolates after treatment of solutions for 10 sec. Figure S2. Confirmation of chemical plaque control agents through LCMS Table S1. Genes specific primers included in this study. [file 12866_2020_1728_MOESM1_ESM.docx]

**Supplementary Figures**

**Supplementary fig. 1:** Growth pattern of *H. pylori* isolates after treatment of solutions for 10 sec.


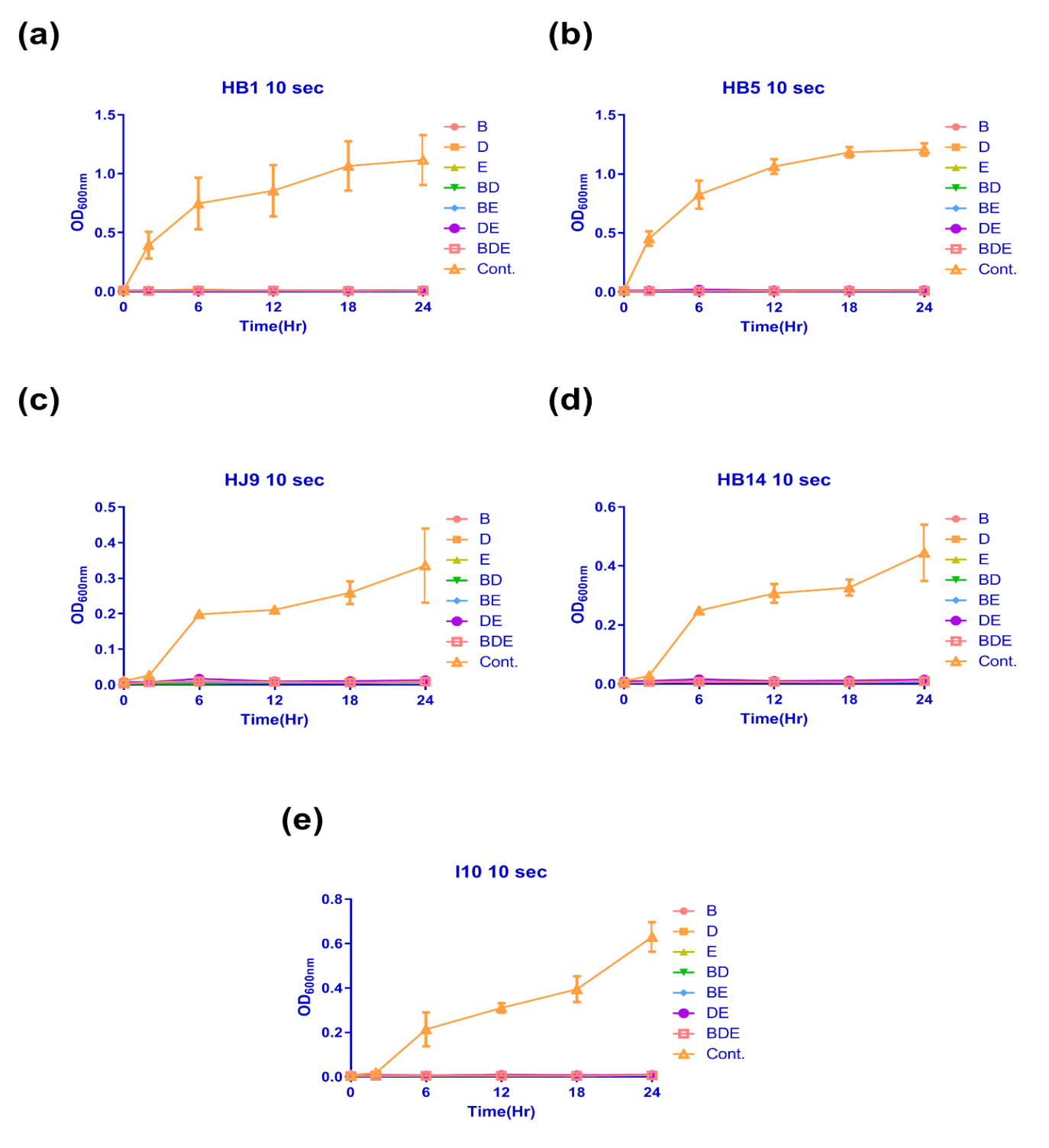


**Supplementary fig. 2:** Confirmation of chemical plaque control agents through LCMS


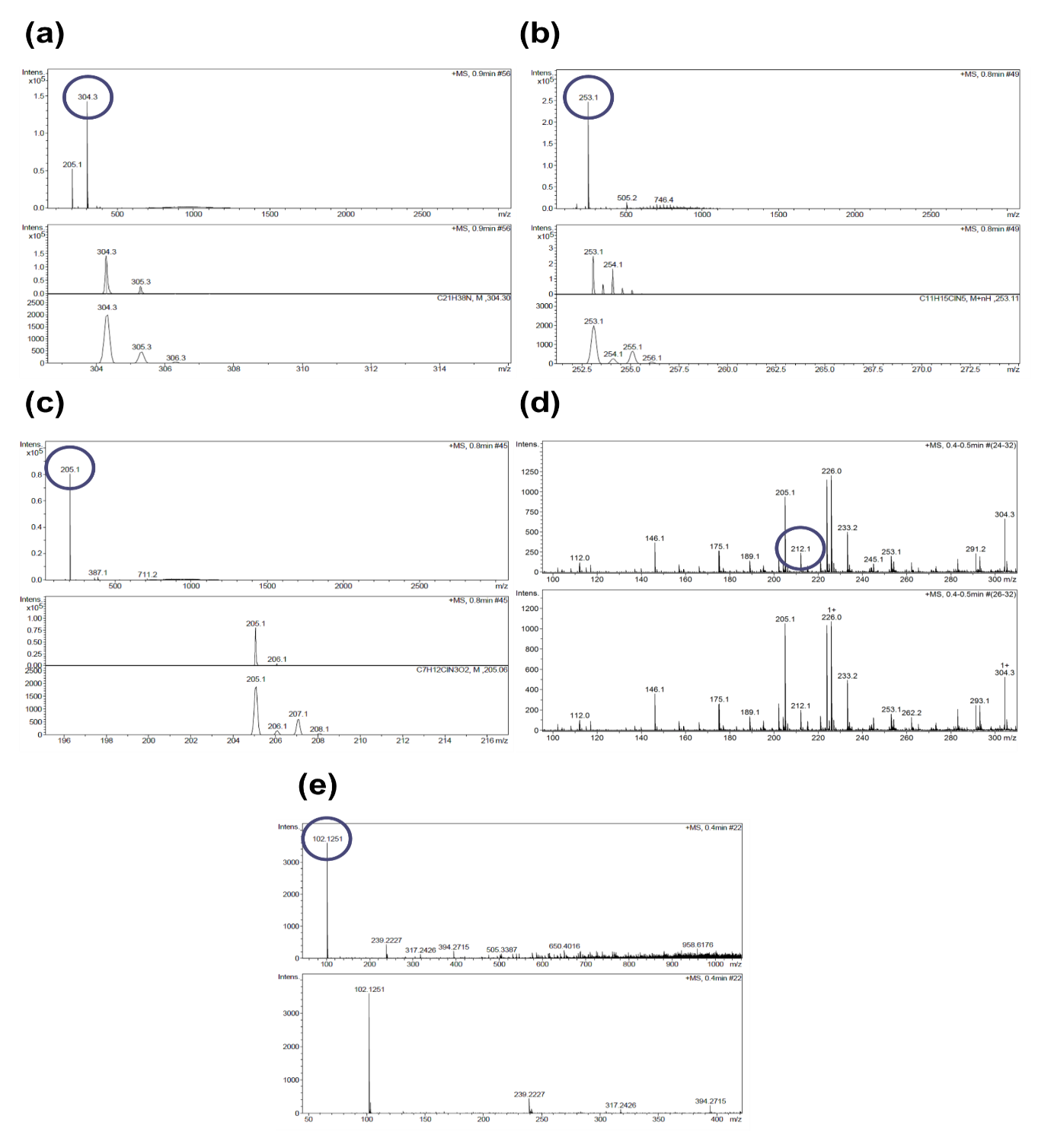


**Supplementary table 1: Genes specific primers included in this study.**

| **Name** | **Sequence (5’-3’)** | **Length**  **(bp)** |
| --- | --- | --- |
| GAPDH-FP | 5′TGCACCACCAACTGCTTAG3′ | 19bp |
| GAPDH-RP | 5′GATGCAGGGATGATGTTC3′ | 18bp |
| CagA-FP | 5′GCCATCATGTTTTAGGCTACC3′ | 21bp |
| CagA-RP | 5′GACGCCCTAGGGAATGATC3′ | 19bp |
| BabA-FP | 5′GATCAACGCGGCGGTAGG3′ | 18bp |
| BabA-RP | 5′CCGTTCAAAGAACAAGTGATGG-3 | 22bp |
| 16SrRNA-FP | 5’CTGGAGAGACTAAGCCCTCC3’ | 21bp |
| 16SrRNA-RP | 5’ATTACTGACGCTGATTGCGC3’ | 21bp |
| APAF1-FP | 5’CTTGCTGCCCTTCTCCATGA3’ | 20bp |
| APAF1-RP | 5’TTGCGAAGCATCAGAATGCG3’ | 21bp |
| FADD-FP | 5’CACCAAGATCGACAGCATCG3’ | 20bp |
| FADD-RP | 5’AGATTCTCAGTGACTCCCGC3’ | 20bp |
| BID-FP | 5’CTGCAGGCCTACCCTAGAGA3’ | 20bp |
| BID-RP | 5’GTGTGACTGGCCACCTTCTT3’ | 20bp |
| BAK-FP | 5’GGTTTTCCGCAGCTACGTTT3’ | 20bp |
| BAK-RP | 5’TAGCGTCGGTTGATGTCGTC3’ | 20bp |
| NOXA-RP | 5’CAAGAACGCTCAACCGAGCC3’ | 20bp |
| NOXA-RP | 5’GCCGGAAGTTCAGTTTGTCTC3’ | 21bp |
| PUMA-FP | 5’GAGCCCGTAGAGGGCCTG3’ | 18bp |
| PUMA-RP | 5’TACTGTGCGTTGAGGTCGTC3’ | 20bp |
| BCL2-FP | 5’CATGTGTGTGGAGAGCGTCA3’ | 20bp |
| BCL2-RP | 5’CATGTAAAGCCAGCCTCCGT3’ | 21bp |
| CDX-2FP | 5’GCAGCCAAGTGAAAACCAGG3’ | 21bp |
| CDX-2RPP | 5’TCTCAGAGAGCCCCAGCG3’ | 20bp |
| CCND1-FP | 5’TGTGCCACAGATGTGAAGTT3’ | 20bp |
| CCND1-RP | 5’CTTGGGGTCCATGTTCTGCT3’ | 20bp |
| PTEN-FP | 5’ACCCACCACAGCTAGAACTT3’ | 20bp |
| PTEN-RP | 5’GGGAATAGTTACTCCCTTTTTGTC3’ | 20bp |
| MMP7-FP | 5’AGTGGTCACCTACAGGATCG3’ | 20bp |
| MMP7-RP | 5’ATCTCCTCCGACCTGTCC3’ | 20bp |
